# Conversation from antiferromagnetic MnBr_2_ to ferromagnetic Mn_3_Br_8_ monolayer with large MAE

**DOI:** 10.1186/s11671-021-03523-0

**Published:** 2021-04-29

**Authors:** Y. Hu, S. Jin, Z. F. Luo, H. H. Zeng, J. H. Wang, X. L. Fan

**Affiliations:** 1 State Key Laboratory of Solidification Processing, Center for Advanced Lubrication and Seal Materials, School of Material Science and Engineering, Northwestern Polytechnical University, 127 YouYi Western Road, Xi’an, 710072 Shaanxi China; 2 Queen Mary University of London Engineering School, Northwestern Polytechnical University, 127 YouYi Western Road, Xi’an, 710072 Shaanxi China

**Keywords:** First-principles calculations, Ferromagnetism, Two-dimensional (2D) materials, Magnetic anisotropy energy (MAE)

## Abstract

A pressing need in low energy spintronics is two-dimensional (2D) ferromagnets with Curie temperature above the liquid-nitrogen temperature (77 K), and sizeable magnetic anisotropy. We studied Mn_3_Br_8_ monolayer which is obtained via inducing Mn vacancy at 1/4 population in MnBr_2_ monolayer. Such defective configuration is designed to change the coordination structure of the Mn-d^5^ and achieve ferromagnetism with sizeable magnetic anisotropy energy (MAE). Our calculations show that Mn_3_Br_8_ monolayer is a ferromagnetic (FM) half-metal with Curie temperature of 130 K, large MAE of − 2.33 meV per formula unit, and atomic magnetic moment of 13/3μ_B_ for the Mn atom_._ Additionally, Mn_3_Br_8_ monolayer maintains to be FM under small biaxial strain, whose Curie temperature under 5% compressive strain is 160 K. Additionally, both biaxial strain and carrier doping make the MAE increases, which mainly contributed by the magneto-crystalline anisotropy energy (MCE). Our designed defective structure of MnBr_2_ monolayer provides a simple but effective way to achieve ferromagnetism with large MAE in 2D materials.

**Supplementary information:**

The online version contains supplementary material available at 10.1186/s11671-021-03523-0.

## Introduction

Spintronics, exploiting the electron spin and the associated magnetic moment, has attracted extensive attention during the past few decades [1], because of its unique advantages over charge-based devices. The recent realization of two-dimensional (2D) ferromagnets with long-range magnetic ordering at finite temperature [2, 3] is of great significance for nanoscale spintronics and related applications and inspires tremendous efforts in investigations and fabrications of 2D ferromagnets [4–9].

The first two 2D ferromagnets with atomic-thickness was achieved in 2017, that is monolayer CrI_3_ [2] and bilayer Cr_2_Ge_2_Te_6_ [3]. Unfortunately, both their Curie temperatures are lower than the liquid-nitrogen temperature (77 K), which limits their realistic applications. Besides the Curie temperature, sizeable magnetic anisotropy and magnetic moment are also indispensable for practical application. Large magnetic anisotropy energy (MAE) implies the benefit for the magnetic ordering against the heat fluctuation, and the possibility to reduce the grain size per bit of information; small MAE may results in super-paramagnetic rather than ferromagnetic. Large magnetic moment provides higher sensitivity, higher efficiency, and higher density for spintronics. Heavy elements are more likely to bring in large MAE due to their strong spin-orbital coupling (SOC) effect [10]. A series of 2D FM materials composed of heavy elements have been predicted having large MAE, such as CrI_3_ [11], CrAs [12], CrSeI [13], CrSiTe_3_ [14], CrWI_6_ [15], FeBr_2_ and FeI_2_ monolayers [16]. Additionally, the local magnetic moment on Mn atom of MXenes Mn_2_NF_2_ and Mn_2_N(OH)_2_ is 4.5μ_B_ per Mn atom [17], which is the largest among the reported FM 2D materials.

Since CrI_3_ monolayer has been successfully synthesized, transition-metal halides have attracted much attentions [18–27]. Spin Seeback effect has been observed in bilayer MnF_2_ [20]; few layers of CrI_3_ has been implemented into the magnetic tunneling junctions (MTJ) [21]; NiCl_3_ monolayer has been predicted to be a novel Dirac spin-gapless semiconductor (SGS) [22]. Particularly, MnBr_2_ monolayer is antiferromagnetic with 0.25 meV MAE along the perpendicular direction to the plane based on the first-principles calculations [16]; Mn^2+^ ions are in the d^5^ high-spin state with magnetic moment of 5μ_B_ [16, 26]. These results imply the potentials of MnBr_2_ as monolayer ferromagnet with large magnetic moment. The key problem is how to convert the AFM coupling between Mn ions into FM coupling.

Significant density of Mn vacancy was observed experimentally in LaMnO_3_ thin films [28], and the concentration of defects can be controlled by regulating the synthesis process deliberately via irradiation of high energy particles, or chemical etching [29]. In this context, we designed the Mn_3_Br_8_ monolayer by inducing single Mn vacancy to MnBr_2_ monolayer. The vacancy changes the coordination structure of the Mn atom and breaks the d^5^ configuration, which may convert the antiferromagnetic coupling into ferromagnetic coupling and bring in large MAE due to the heavy Br atom. As we expect, Mn_3_Br_8_ monolayer is FM and has large MAE of − 2.33 meV per formula unit, the magnetic moment for each Mn atom is 13/3μ_B_. Considering the easy introducing of strain via bending flexible substrates [30–33], elongating elastic substrate [33–35], exploiting the thermal expansion mismatch [33, 36], and so on [33], and the effective control of spin polarization via electrostatic doping [37, 38], we also studied the Mn_3_Br_8_ monolayer under biaxial strain and carrier doping. Our results show that Mn_3_Br_8_ monolayer maintains to be FM with Curie temperature increasing under small biaxial strain. Plus, both biaxial strain and carrier doping can make the MAE increases.

## Computational methods

All the calculations in the present study were performed by adopting the spin-polarized density function theory (DFT) method as implemented in the Vienna *ab-initio* simulation package (VASP) [39]. Interactions between electrons and nuclei were described by the projector augmented wave (PAW) method [40, 41], and the electronic exchange–correlation interactions were described by the Perdew–Burke–Ernzerhof (PBE) functional within the generalized gradient approximation (GGA) method [42]. The Hubbard U terms were adopted to calculate the strong-correlated interaction [43]; an effective on-site coulomb interaction parameter (U) of 4 eV and an exchange energy (J) of 1 eV which was adopted for studying Mn-incorporated 2D materials were used for the Mn-d electrons [44]. The Brillouin zone integration was carried out by adopting the 9 × 9 × 1 k-mesh based on the Monkhorst–Pack scheme [45]. The phonon spectrums were calculated using the Phonopy code [46] which is implemented within the VASP package. A vacuum space of 20 Å was added along the direction perpendicular to the surface of the monolayer to avoid the interaction between the adjacent layers. The cutoff energy for the plane wave basis set was set as 500 eV. The convergence criterion for the total energy and force was set as 1 × 10^–6^ eV and 0.01 eV/Å, respectively.

## Results and discussions

### Cleavage energy, ground state, and stability of the MnBr_2_ monolayer

**Fig. 1:**
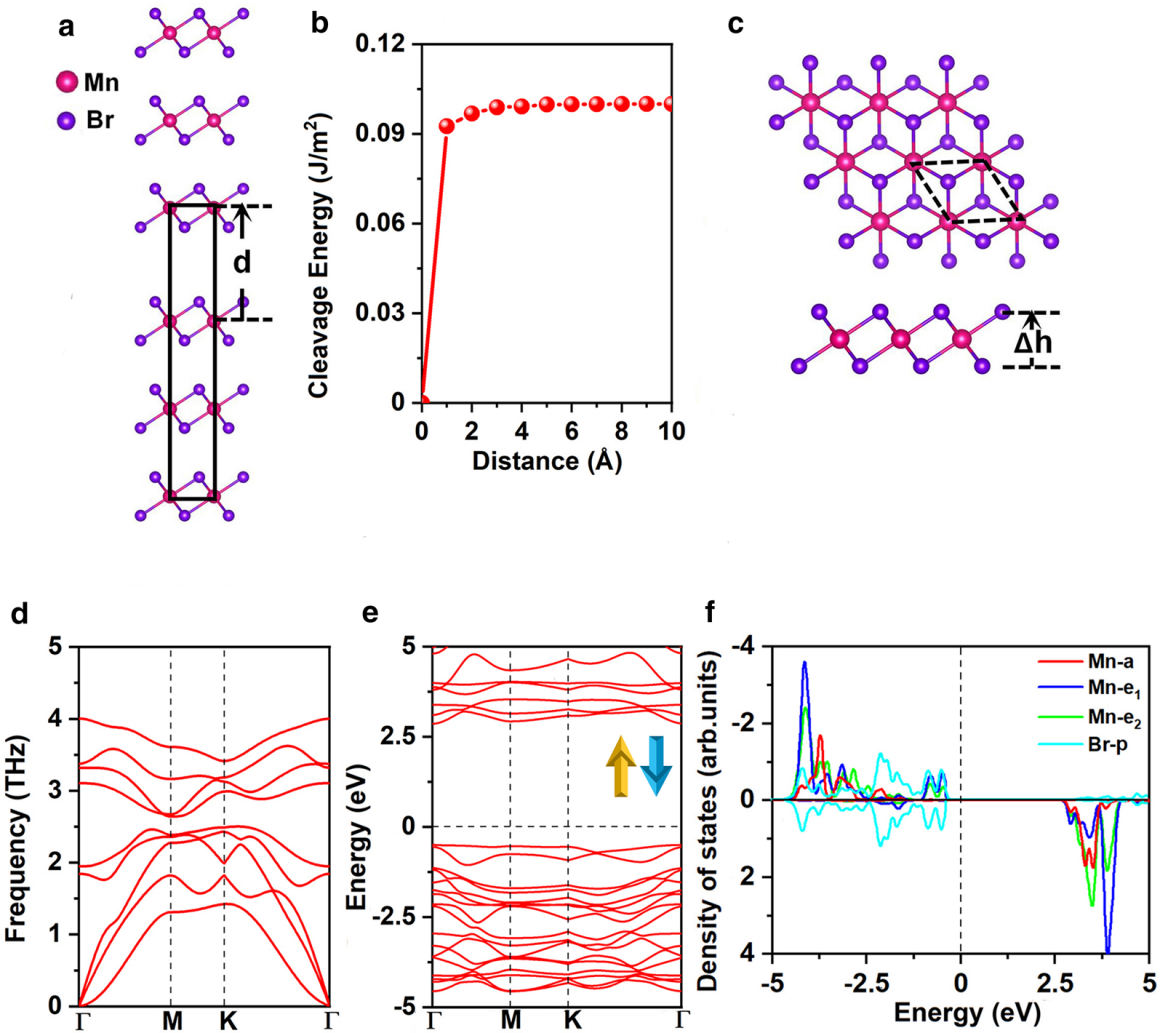
**a** Bulk model of MnBr_2_ used to calculate the cleavage energy and **b** the cleavage energy as a function of the separation distance $d$ between two fractured parts (the equilibrium interlayer distance is set as 0). **c** Top and side views, **d** phonon spectrum, **e** electronic band structure for both spin channels and **f** projected density of states (PDOS) of Mn-d orbitals and Br-p orbitals for MnBr_2_ monolayer. Δ*h* represents the vertical distance between two halide planes. The primitive cell is circulated in black dash lines. The Fermi level for band structure and DOS is set as 0 eV

The optimized lattice constants of bulk MnBr_2_ are a = b = 3.95 Å, consistent with the previous experimental result (*a* = *b* = 3.87 Å) [25]. We firstly explored the feasibility of exfoliating MnBr_2_ monolayer from the bulk MnBr_2_. Figure 1a presents the well-known, effective, and widely approved method of calculating the cleavage energy [47–49]. Specifically, the cleavage energy was obtained by calculating the variation of the total energy of the ground state with respect to the separation distance $d$ between the two fracture parts as shown in Fig. 1b, the lattice constants of a and b are fixed as the values at the equilibrium state of bulk MnBr_2_. The interlayer long-range vdW interactions was described by the Grimme’s DFT-D2 scheme [50, 51]. The total energy increases with separation distance and then slowly converges as shown in Fig. 1b. The calculated cleavage energy is 0.10 J/m^2^, which is smaller compared with the cleavage energy between the two fracture parts of graphite (0.35 J/m^2^) [52], demonstrating the feasibility of obtaining MnBr_2_ monolayer via micro-mechanical exfoliating method.

**Fig. 2:**
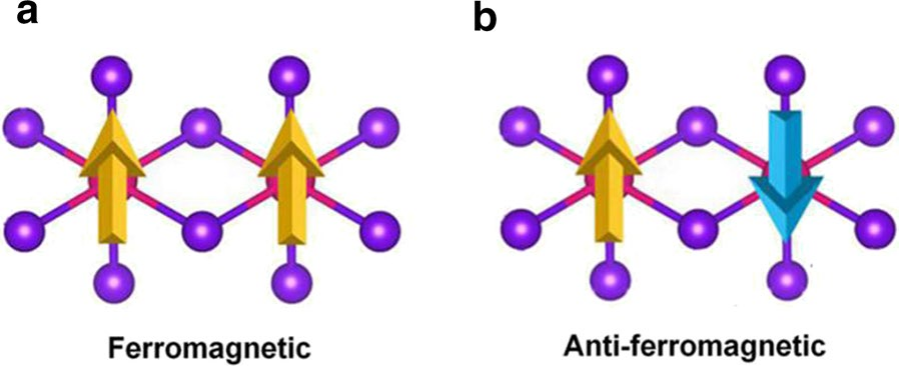
Schematic diagrams for **a** ferromagnetic and **b** antiferromagnetic configurations for MnBr_2_ monolayer

MnBr_2_ monolayer has the $C_{{{3}v}}$ symmetry as shown in Fig. 1c; each Mn atom is surrounded by 6 neighboring Br atoms, forming an octahedral [MnBr_6_]^4−^ unit. As shown in Fig. 2a and b, three possible magnetic configurations, namely non-magnetic (NM), ferromagnetic (FM), and antiferromagnetic (AFM) states are considered. Both high-spin and low-spin states of the Mn ion are considered. Our results show that the Mn ions of FM state are in low-spin with d^1^ configuration, while the Mn ions in AFM state are in high-spin with d^5^ configuration. The ground state of MnBr_2_ monolayer is the AFM state, which is more stable than the NM and FM states by 3.91 eV and 0.72 eV per formula unit, respectively (Additional file 1: Table. S1). The MAE is 0.25 meV, the positive value indicating that the easy magnetization axis is along the out-of-plane directions, agreeing with the previous result [16]. The optimized lattice constants are *a* = *b* = 3.95 Å, same with the lattice constants of the bulk MnBr_2_. The Mn-Br bond length is 2.73 Å, and the vertical distance between the two halide planes is 3.03 Å.

The stability of the MnBr_2_ monolayer was further investigated by calculating the formation energy, phonon spectrum, and elastic constants. The formation energy is calculated as:

$$E_{{{\text{form}}}} = E_{{{\text{MnBr}}_{{2}} }} - E_{{{\text{Mn}}}} - 2E_{{{\text{Br}}}}$$

where $E_{{{\text{MnBr}}_{{2}} }}$ represents the energy of MnBr_2_ monolayer, $E_{{{\text{Mn}}}}$ and $E_{{{\text{Br}}}}$ are the energies of Mn and Br atoms in their bulk structures, respectively. The calculated $E_{{{\text{form}}}}$ is − 1.87 eV per atom; the negative value means that the formation is exothermic and MnBr_2_ monolayer is energetical favorable. Plus, our calculated phonon spectrum (Fig. 1d) for MnBr_2_ monolayer shows no negative frequency in the whole Brillouin zone, indicating dynamically stable. Additionally, the calculated elastic constants (Additional file 1: Table S2) comply with the Born-Huang criteria [53] of $C_{11} > 0$, $C_{11} C_{22} - C_{12}^{2} > 0$ and $C_{66} > 0$, confirming that MnBr_2_ monolayer is mechanically stable. The calculated in-plane stiffness is 26.98 J/m^2^, about 75% of the MnPSe_3_ (36 J/m^2^) [49], and 15% of MoS_2_ monolayer (180 J/m^2^) [54]. Plus, MnBr_2_ monolayer demonstrates higher flexibility, and the ability of sustaining larger tensile strain comparing with MoS_2_ monolayer (11%) [54]. This may attributes to ionic bonds for MnBr_2_ monolayer against the covalent bonds of MoS_2_ monolayer. The analysis of the deformation related to elastic constants indicates it can withstand its weight (See details in the SI).

The electronic band structure of MnBr_2_ monolayer is shown in Fig. 1e, it indicates that MnBr_2_ monolayer is a semiconductor with a direct band gap of 3.35 eV. Both valence band maximum (VBM) and conduction band minimum (CBM) are located at the $\Gamma$ point. To gain insight of the electronic structures, projected density of states (DOS) for the Mn-d and Br-p orbital are presented in Fig. 1f. The five d orbitals of Mn ion split into $a(d_{{z^{2} }} )$, $e_{1} (d_{xz} + d_{yz} )$, and $e_{2} (d_{xy} + d_{{x^{2} - y^{2} }} )$ groups according to the $C_{{{3}v}}$ symmetry. The bader charge analysis suggests that each Mn atom donates two electrons to the two neighboring Br atoms. Thus, the five d-orbitals in one spin-channel are fully occupied by the five d-electrons of the Mn^2+^ ions. Correspondingly, the two Mn^2+^ ions in the unit cell are in the d^5^ high-spin state with the magnetic moment of 5μ_B_/− 5μ_B_, the Br^1−^ ions are in the low-spin state of 4p^6^ with neglectable magnetic moment of − 0.02μ_B_ (Additional file 1: Fig. S1(a)). According to the Goodenough–Kanamori–Anderson (GKA) rule, such configuration always provides antiferromagnetic coupling [55].

### Stability, electronic, and magnetic properties of Mn_3_Br_8_ monolayer

**Fig. 3:**
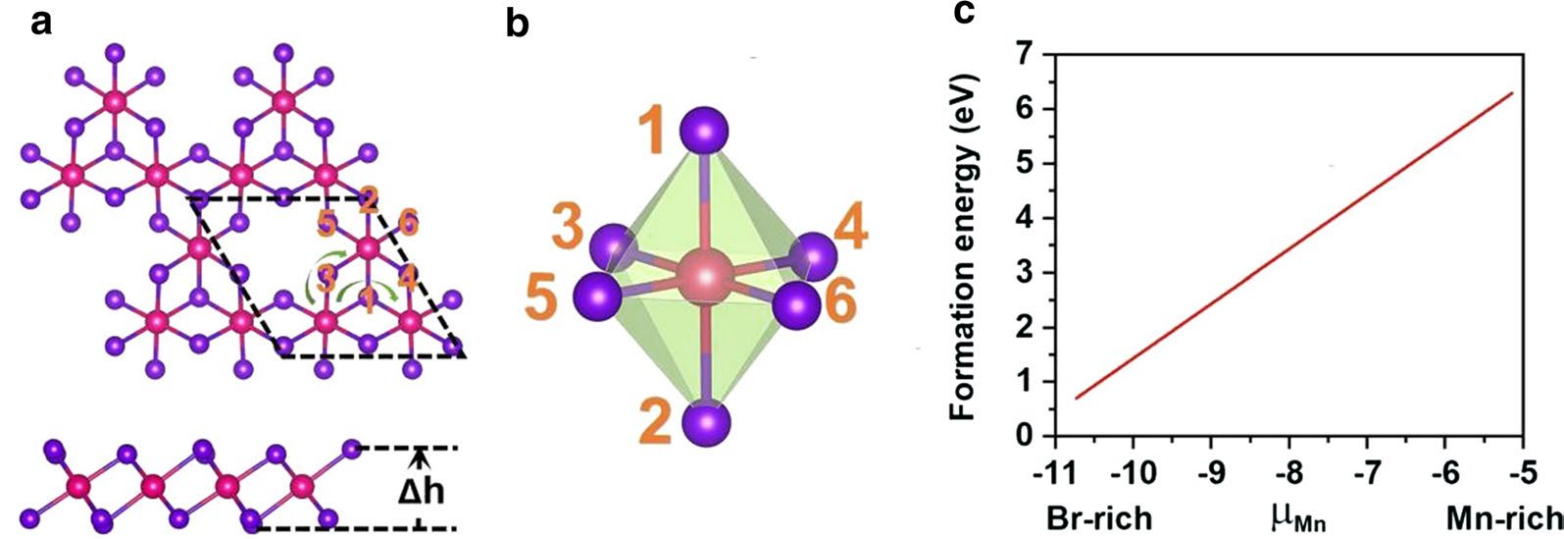
**a** Top and side views of Mn_3_Br_8_ monolayer, $\Delta h$ represents the vertical distance between two halide planes. The primitive cell is circulated in black dash lines; the green arrow lines show two different paths of the super-exchange interaction. **b** Structure of the distorted MnBr_6_ octahedron. **c** Formation energies for single Mn vacancy as a function of chemical potential of Mn (μMn)

**Fig. 4:**
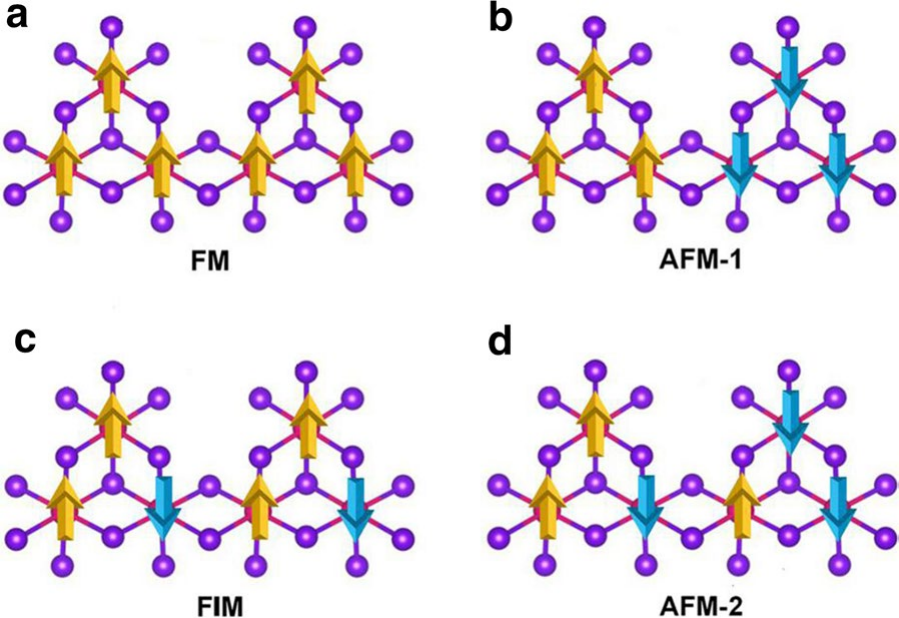
Schematic diagrams for **a** ferromagnetic, **b** antiferromagnetic-1, **c** ferrimagnetic,and **d** antiferromagnetic-2 configurations for Mn_3_Br_8_ monolayer

Mn vacancy was introduced to break the d^5^ configuration of the Mn^2+^ ions. Single Mn vacancy is introduced in the $2 \times 2 \times 1$ supercell of MnBr_2_ monolayer, which gives out the Mn_3_Br_8_ monolayer. As shown in Fig. 3a, each Mn atom has four nearest neighboring Mn atoms and binds to six Br atoms, forming a distorted octahedral [MnBr_6_] unit. Five magnetic states (NM, FM, FIM, AFM-1, and AFM-2) shown in Fig. 4 were considered. Our results indicate that the FM state is the ground state, which is more stable than the other four by 9.84 eV, 32.90 meV, 129.85 meV, and 97.65 meV per formula unit, respectively. The optimized lattice constant is still 3.95 Å. Different from MnBr_2_ monolayer, Mn_3_Br_8_ monolayer has 2 types of Mn-Br bonds (Fig. 3b). The bonds between Mn atom and the two central Br atoms ($d_{{\text{Mn-Br1,2}}}$) are 2.76 Å, while the other Mn–Br bonds ($d_{{\text{Mn-Br3,4,5,6}}}$) are 2.59 Å. The vertical distance between the two halide planes is 3.33 Å.

**Fig. 5:**
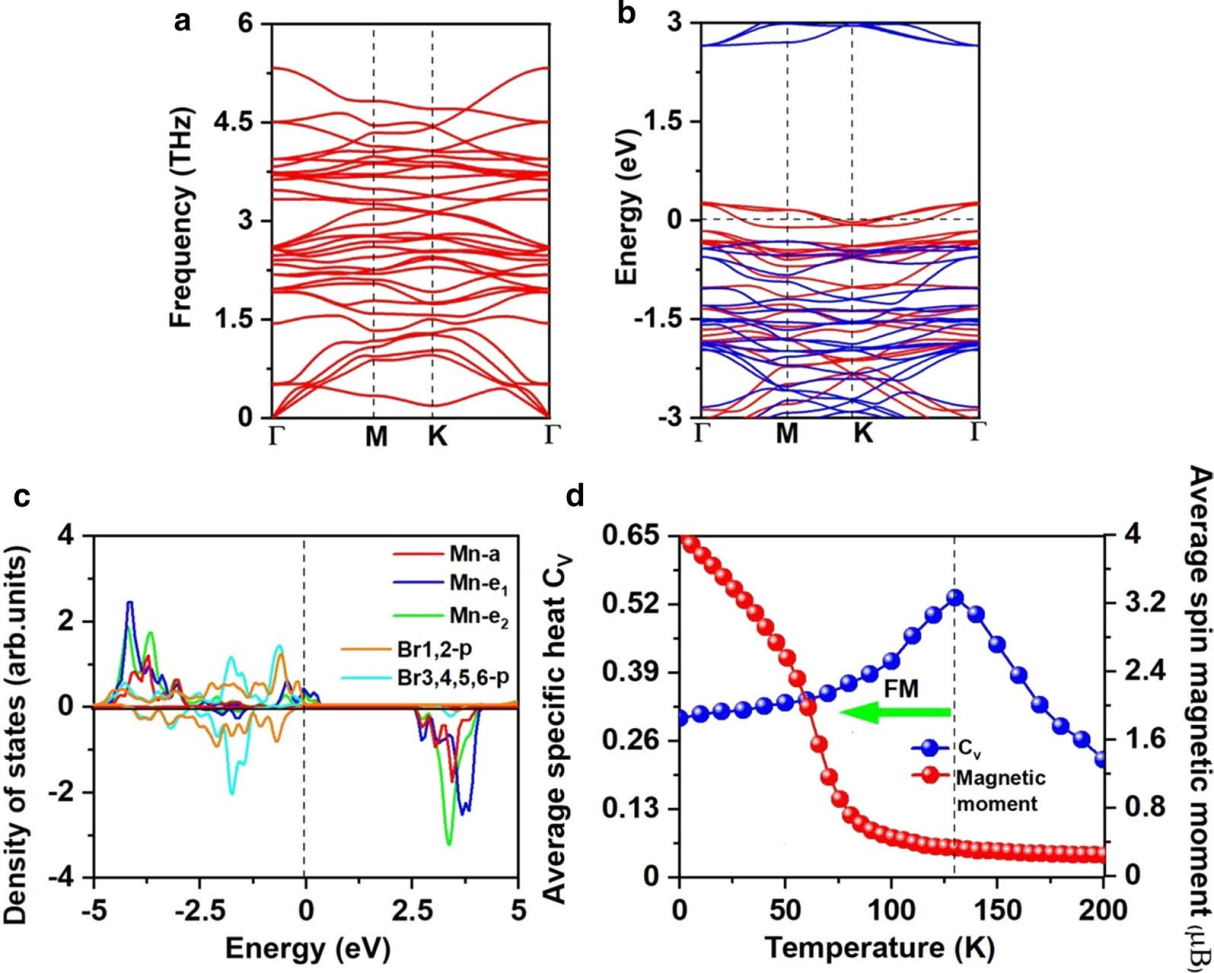
**a** Phonon spectrums, **b** spin-resolved electronic band structure, and **c** projected density of states (PDOS) of Mn-d orbitals and Br-p orbitals for Mn_3_Br_8_ monolayer. **d** On-site magnetic moments of Mn atoms and the specific heat *C*_*v*_ as function of temperature based on Heisenberg model for Mn_3_Br_8_ monolayer. The Fermi level for band structure and PDOS is set as 0 eV

To verify the feasibility of inducing Mn vacancy, we firstly calculated the vacancy formation energies under Mn-rich and Br-rich environments via following equations,

$$E_{{F({\text{Mn-rich}})}} {\text{ = }}E_{{{\text{Mn}}_{3} {\text{Br}}_{8} }} - (4 \times E_{{{\text{MnBr}}_{{\text{2}}} }} - \mu _{{{\text{Mn-max}}}} )$$

$$E_{{F{\text{(Br-rich)}}}} { = }E_{{{\text{Mn}}_{{3}} {\text{Br}}_{{8}} }} - (4 \times E_{{{\text{MnBr}}_{{2}} }} - \mu_{{\text{Mn-min}}} )$$

where $E_{{{\text{Mn}}_{{3}} {\text{Br}}_{{8}} }}$ and $E_{{{\text{MnBr}}_{{2}} }}$ represent the total energies of the Mn_3_Br_8_ and MnBr_2_ monolayers, $\mu_{{\text{Mn-max}}}$ is the chemical potential of Mn under Mn-rich environment, which is calculated as the energy of Mn atom in its bulk structure, $\mu_{{\text{Mn-min}}}$ is the chemical potential of Mn under the Br-rich environment, which is calculated as:

$$\mu_{{\text{Mn-min}}} = E_{{{\text{MnBr}}_{{2}} }} - 2 \times \mu_{{\text{Br-max}}}$$

where $\mu_{{\text{Br-max}}}$ is the chemical potential of Br and calculated as the energy of Br atom in gas phase. As shown in Fig. 3c, the formation energies under Mn-rich/Br-rich environment are 6.30/0.71 eV per Mn vacancy, indicating that the formation of Mn vacancy is energetically more favorable under the Br-rich environment. Indeed, the S vacancy has been experimentally achieved in MoS_2_ monolayer [56], and the predicted formation energy of S vacancy under the S-rich environment is 2.35 eV [57]. Moreover, structuring porous nano-architecture like β-FeOOH/PNGNs (porous nitrogen-doped graphene networks) can induce significant Fe-vacancy [58], and the Bridgman method was adopted to induce ordering Fe vacancy. We also hope that these methods are applicable for inducing Mn vacancy [59]. Plus, there is no negative frequency found in the phonon spectrum of Mn_3_Br_8_ monolayer shown in Fig. 5a, proving the dynamical stability. These results approve our design of introducing Mn vacancy to bring in ferromagnetism.

The ferromagnetism of Mn_3_Br_8_ monolayer attributes to the FM super-exchange interaction. According to the Goodenough–Kanamori–Anderson (GKA) rule [55], super-exchange interaction between the Mn ions is FM when the Mn-Br-Mn angle is around 90°. In such configuration (Additional file 1: Fig. S2), the Mn-d orbital tends to AFM couples with different orthogonal Br-p orbitals, and thus the indirect Mn–Mn magnetic coupling is expected to be FM. But if each Mn ion has 5 unpaired electrons like MnBr_2_ monolayer, super-exchange is AFM although the Mn-Br-Mn angle is close to 90° because there are no empty spin-up Mn-d orbitals left in MnBr_2_ monolayer and spin-up d-electrons cannot hop between the neighboring Mn site [60]. There are existing two different super-exchange interaction paths in Mn_3_Br_8_ (Fig. 3a), and both are FM. One involves central Br1,2 atoms with Mn-Br bond lengths of 2.76 Å and Mn-Br-Mn angles of 87.5°; the other one involves Br3,4,5,6 atoms with Mn-Br bond length of 2.59 Å and Mn-Br-Mn angles of 95°. The hybridized interactions between p orbitals of Br3,4,5,6 atoms and Mn-d orbitals are stronger than that of p-d hybridization involving Br1,2 atoms, as shown in Fig. 5c, particularly from − 2 eV to − 1.4 eV. While from 1.4 to − 0.9 eV, the *p*-*d* hybridization involving Br1,2 atoms are dominated.

The bader charge analysis suggests that each Mn atom donates 8/3 electrons to the neighboring Br atoms. Thus, the Mn ions are in the Mn^8/3+^ state. As shown in Fig. 5c, the 13/3 electrons of each Mn ion all fill in the spin-up channel of the d-orbital, while the Br^1−^ ions are in the low-spin state of 4p^6^. Thus, the magnetic moment of each Mn^8/3+^ ion is 13/3μ_B_; the magnetic moment of Br^1−^ ions are neglectable (Additional file 1: Fig. S1(b)). Inducing ferromagnetism by vacancy can also be observed for the d^0^ systems, like ZnS and ZnO [61, 62], single vacancy can induce magnetic moment as large as 2μ_B_ [61]_._ For each Mn ion, 2/3 d-orbital is unoccupied; the spin-up channel of both $e_{1}$ and $e_{{2}}$ orbitals are partially occupied and crossing the Fermi level, resulting in half-metallicity. The half-metallic character also can be observed from the spin-resolved electronic band structure shown in Fig. 5b. The spin-up channel is metallic, while the spin-down channel is semiconducting with the indirect band gap of 2.97 eV; the VBM/CBM locates at the ${\text{M}}$/$\Gamma$ point. The value of the band gap is close to those of the MnP (2.86 eV) [63], MnAs (2.92 eV) [63], and Ni_2_NO_2_ (2.98 eV) [64], which is large enough to prevent the thermally excited spin-flip. Comparing with the MnBr_2_ monolayer, both the VBM and CBM of the semiconducting channel get more closer to the Fermi level. The CBM is still dominated by the Mn atoms, while the VBM is dominated by the new Br1,2 atoms. Meanwhile, the semiconducting channel converts from direct to indirect, and the band gap reduces. The similar phenomenon was observed in MnCl_2_ monolayer with H functionalization [60].

The magnetization directions are determined by the magnetic anisotropy energy (MAE). The MAE of solids arises from two contributors, namely the magneto-crystalline energy (MCE) related to the spin–orbit coupling (SOC) and the magnetic dipolar anisotropy energy (MDE) attributed by the magneto-static dipole–dipole interaction. The MDE in the 3D isotropic materials, such as bcc Fe and fcc Ni, is very small. But for low-dimensional materials composed of transition metal atoms with large magnetic moment, the MDE should not be ignored [65–67]. The MCE is defined as the difference between the magnetization energy along the in-plane (100 or 010) and out-of-plane (001) directions by taking the SOC into account. The MDE is obtained as the difference of $E_{d}$ between the in-plane and out-of-plane magnetizations. $E_{d}$ in atomic Rydberg units is given by [65, 66]

$$E_{d} = \sum\limits_{ij} {\frac{{2m_{i} m_{j} }}{{c^{2} }}} M_{ij}$$

where the speed of light, $c = 274.072$, $i/j$ are the atomic position vectors in the unit cell, and ${m}_{i}/{m}_{j}$ is the atomic magnetic moment (μ_B_) on site $i/j$. The magnetic dipolar Madelung constant $M_{ij}$ is calculated via

$$M_{ij} = \sum\limits_{R} {\frac{1}{{\left| {R + i + j} \right|^{3} }}} \left\{ {1 - 3\left. {\frac{{\left[ {(R + i + j) \cdot \mathop {m_{i} }\limits^{ \wedge } } \right]^{2} }}{{\left| {R + i + j} \right|^{2} }}} \right\}} \right.$$

where $R$ are the lattice vectors. In a 2D material, since all the $R$ and $i$ are in-plane, the second term would be zero for the out-of-plane magnetization, resulting in the positive $M_{ij}$, while $M_{ij}$ is negative for an in-plane magnetization [67]. Therefore, the MDE relates to the magnetic moment of transition metal and always prefers the in-plane magnetization.

**Fig. 6:**
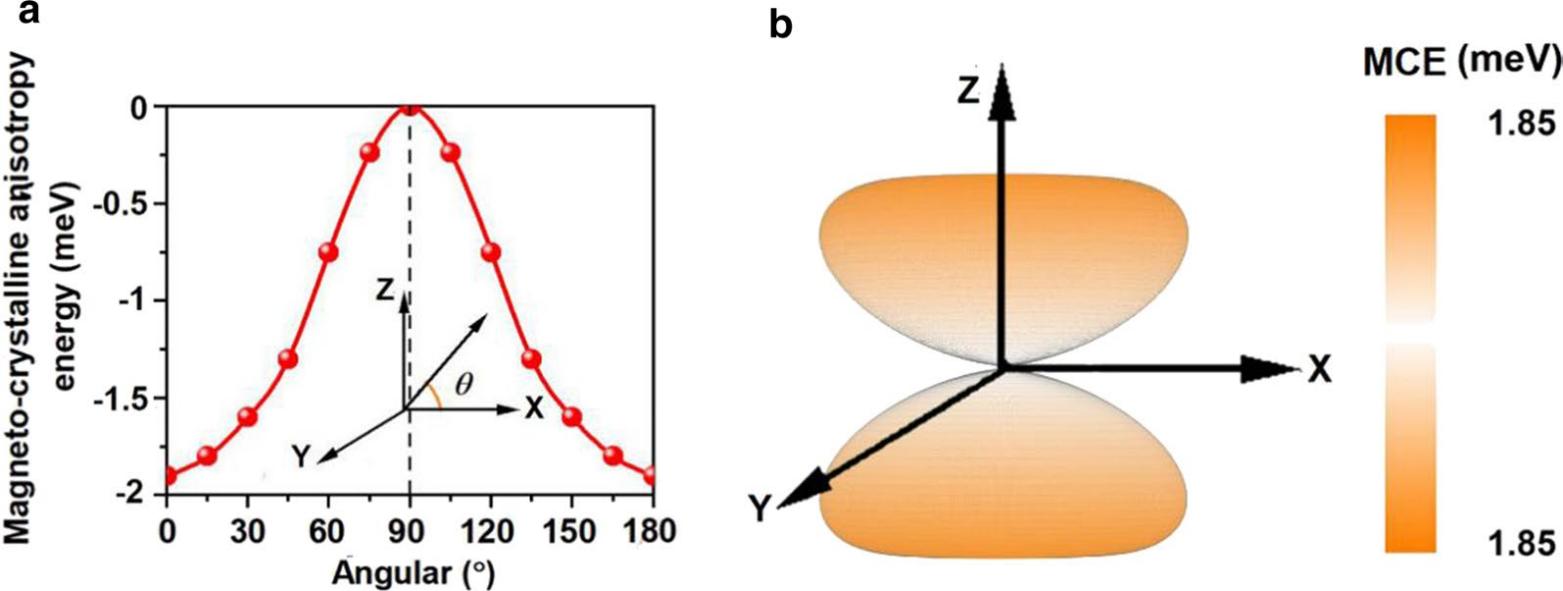
Variation of magneto-crystalline anisotropy energy (MCE) **a** with respect to azimuthal angle and **b** in the space for Mn_3_Br_8_ monolayer

The calculated MCE for Mn_3_Br_8_ monolayer is − 1.90 meV per formula unit (Fig. 6a), much larger than those of bulk Fe (0.001 meV per atom), and Ni (0.003 meV per atom) [68], and larger than that of the Fe monolayer on Rh (111) (0.08 meV per atom) [69], suggesting that the magnetization of the Mn_3_Br_8_ monolayer is thermal stable. The relationship between the MCE and the azimuthal angle can be described by the following equation [70]:

$${\text{MCE}}(\theta ) = A\cos^{2} (\theta ) + B\cos^{4} (\theta )$$

where $A$ and $B$ are the anisotropy constants and $\theta$ is the azimuthal angle. The fitting result is shown in Additional file 1: Figs. S3. Additionally, the evolution of MCE with the spin axis rotating through the whole space is illustrated in Fig. 6b. MCE within the xy plane shows no difference, but reaches the maximum value along the direction perpendicular to the xy plane, confirming the strong magnetic anisotropy. The MDE is − 0.43 meV per formula unit, and MAE (MCE + MDE) is − 2.33 meV per formula unit. The negative value indicates that the easy magnetization axis is along the in-plane directions. The MDE does not change the magnetization direction, but enhances it. Additionally, the MAE of Mn_3_Br_8_ monolayer is much larger than that of MnBr_2_ monolayer, proving again the effectiveness of our design.

We further calculated the $T_{c}$ for FM Mn_3_Br_8_ monolayer by performing the Monte Carlo (MC) simulations based on the Heisenberg model, which has been proven to be the effective method for predicting $T_{c}$ for 2D materials [11, 15, 48, 58, 71–76]. Our estimated $T_{c}$ of CrI_3_ monolayer is 42 K (Additional file 1: Fig. S4) [76], agreeing well with the experimental measured value [2] and previous calculation results [15, 58, 71, 72, 74, 76], which proves the accuracy of our adopted method. The spin-Hamiltonian including the nearest neighboring (NN) magnetic interaction is described as

$$H = - \sum\limits_{i,j} {JM_{i} M_{j} }$$

where $J$ is the NN magnetic exchange parameter, $M_{i/j}$ is the magnetic moment of Mn ions and integral close to the number of the spin polarized electrons based on Monte Carlo method [71, 77, 78], $i$ and $j$ stand for the NN pair of Mn ions. The magnetic coupling parameter $J$ is calculated via the energy difference between the FM and AFM states as

$$J{ = }\frac{{E_{{{\text{AFM1}}}} - E_{{{\text{FM}}}} }}{{16M^{2} }}$$

The calculated $J$ of NN Mn ions is 1.01 meV; the positive value indicates the preferring of FM coupling.

The calculated $J$ of the NN Mn ions and the $100 \times 100 \times 1$ supercell containing 20,000 magnetic moment vectors were adopted to perform the MC simulations. The simulations at each temperature lasts for 10^5^ steps. Each magnetic moment vector rotates randomly in all directions. Figure 5d shows the evolution of specific heat defined as $C_{{_{V} }} = {{\left( {\left\langle {E^{2} } \right\rangle - \left\langle E \right\rangle^{2} } \right)} \mathord{\left/ {\vphantom {{\left( {\left\langle {E^{2} } \right\rangle - \left\langle E \right\rangle^{2} } \right)} {K_{B} T^{2} }}} \right. \kern-\nulldelimiterspace} {K_{B} T^{2} }}$ with temperature, from which we obtained the $T_{c}$ of 130 K for Mn_3_Br_8_ monolayer by locating the peak position of $C_{v}$, higher than the liquid-nitrogen temperature (77 K), and $T_{c}$ of CrI_3_ (45 K) [2] and Cr_2_Ge_2_Te_6_ (28 K) [3], CrX_3_ (X = F, Cl, Br) (36 ~ 51 K) [11], CrXTe_3_ (X = Si, Ge) (35.7 K, 57,2 K) [48]. Our calculations demonstrate that the FM Mn_3_Br_8_ monolayer has the large MAE and Curie temperature higher than the liquid-nitrogen temperature.

## Mn_3_Br_8_ monolayer under biaxial strain and carrier doping

**Fig. 7:**
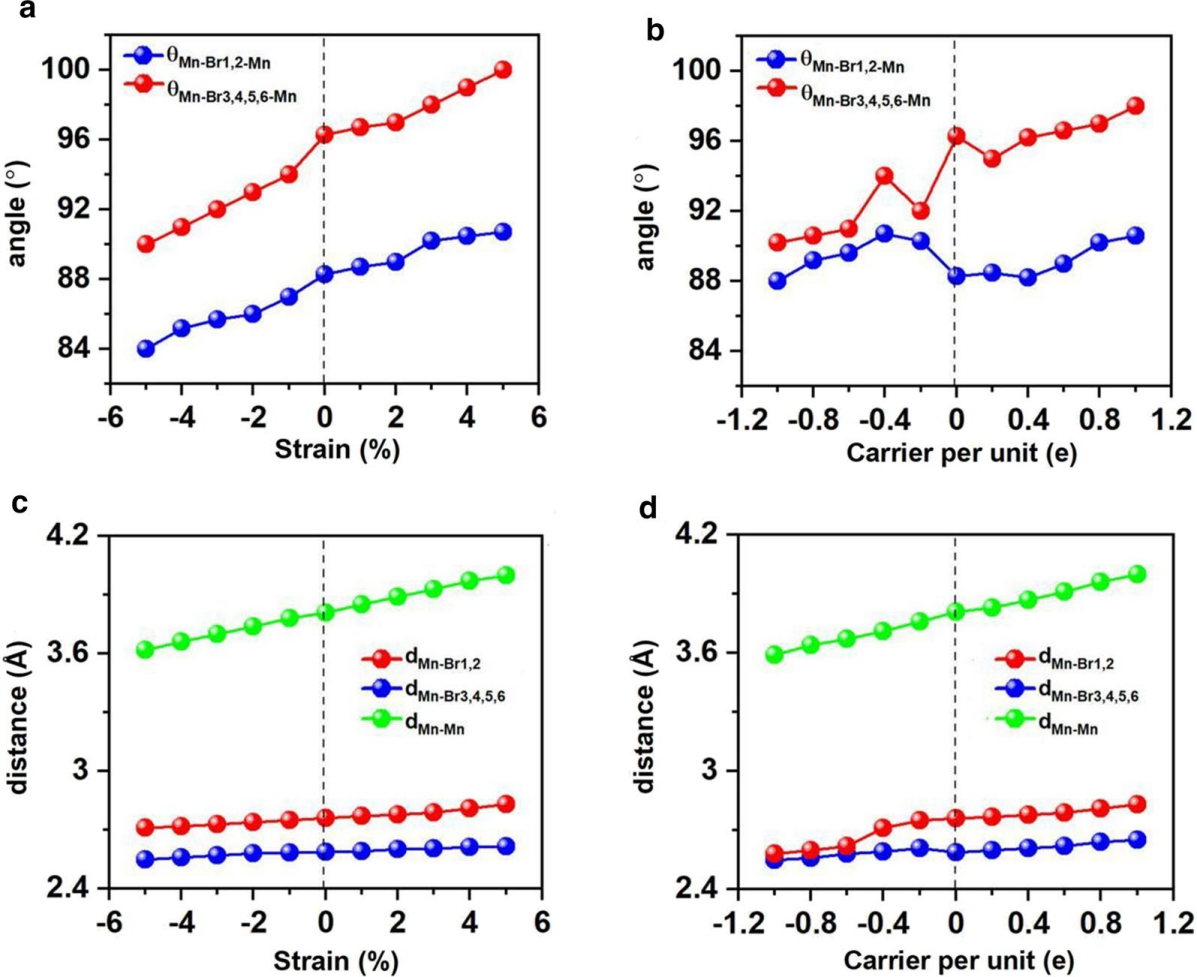
Variations of angles between two Mn and Br atoms, the distance between Mn and Br atoms, and distance between nearest neighboring Mn atoms with respect to the applied biaxial strain and carrier doping. Variation of **a** angle and **c** distance with respect to biaxial strain, variations of **b** angle and **d** distance with respect to carrier doping. Positive and negative values of carrier doping represent the electron and hole doping, respectively

Strain engineering has been proven applicable for many 2D materials, and effective to alter the structural parameters, such as the bond lengths and angles, and tune the electronic and magnetic properties. In this context, we investigated Mn_3_Br_8_ monolayer under the biaxial strain ranging from − 5% to 5%. It turns out that Mn_3_Br_8_ monolayer under biaxial strain from − 5 to 5% maintains to be FM and the atomic magnetic moment hardly changes. As shown in Figs. 7a and c, the angles between two Mn atoms and Br1,2 atoms (θ_Mn-Br1,2-Mn_) are 84°–90°, which increases as the strain and gradually approaches 90°. The Mn–Br–Mn angles involving Br3,4,5,6 atoms (θ_Mn-Br3,4,5,6-Mn_) gradually deviate from 90°, ranging from 90° to 100°. Thus, super-exchange interactions between the Mn ions mediated via different orthogonal Br-p orbitals are still FM.

**Fig. 8:**
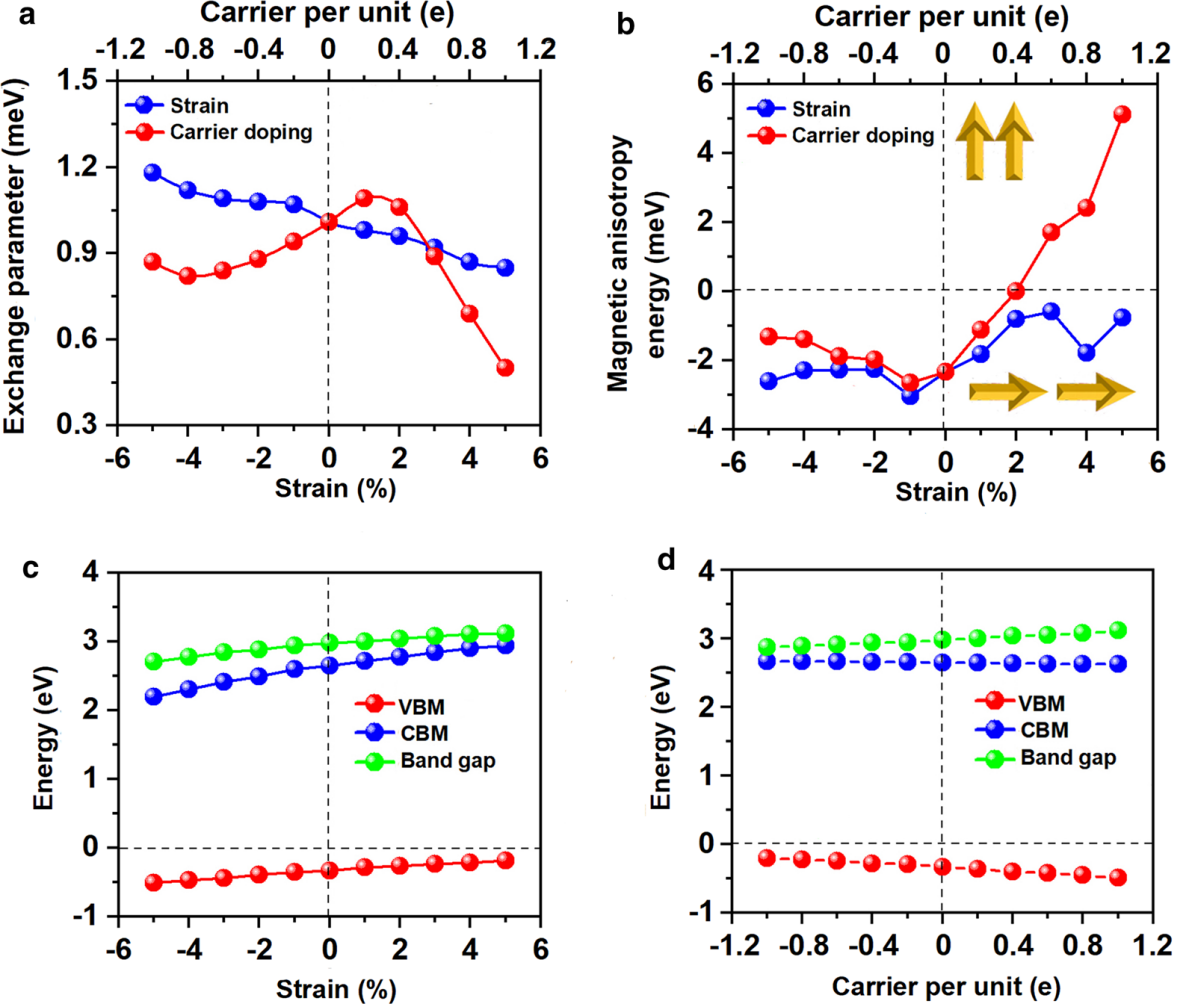
Variations of **a** the exchange parameter and **b** magnetic anisotropy energy (MAE) for Mn_3_Br_8_ monolayer with respect to the applied biaxial strain and carrier doping. The variations of valence band maximum (VBM), conduction band minimum (CBM), and band gap in the semiconducting channel for Mn_3_Br_8_ monolayer with respect to **c** the applied biaxial strain and **d** carrier doping ranging. Positive and negative values of the carrier doping represent the electron and hole doping, respectively

**Fig. 9:**
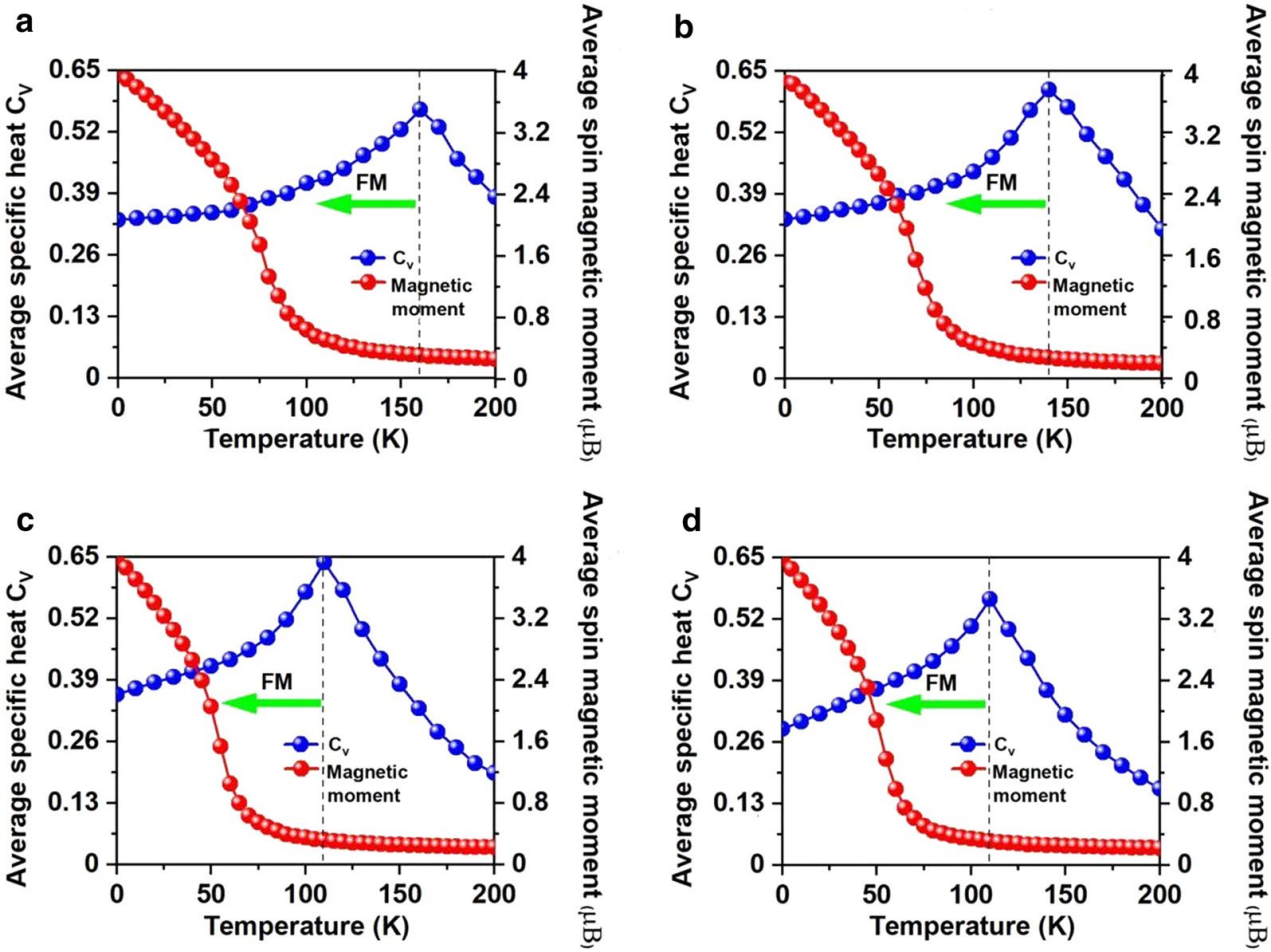
On-site magnetic moments of Mn atoms and the specific heat *C*_*v*_ as function of temperature based on Heisenberg model for Mn_3_Br_8_ monolayer **a** under -5% biaxial strain, with **b** 0.2e, **c** -0.6e, and **d** -0.8e carrier doping per formula unit. Positive and negative values represent the electron and hole doping, respectively

**Fig. 10:**
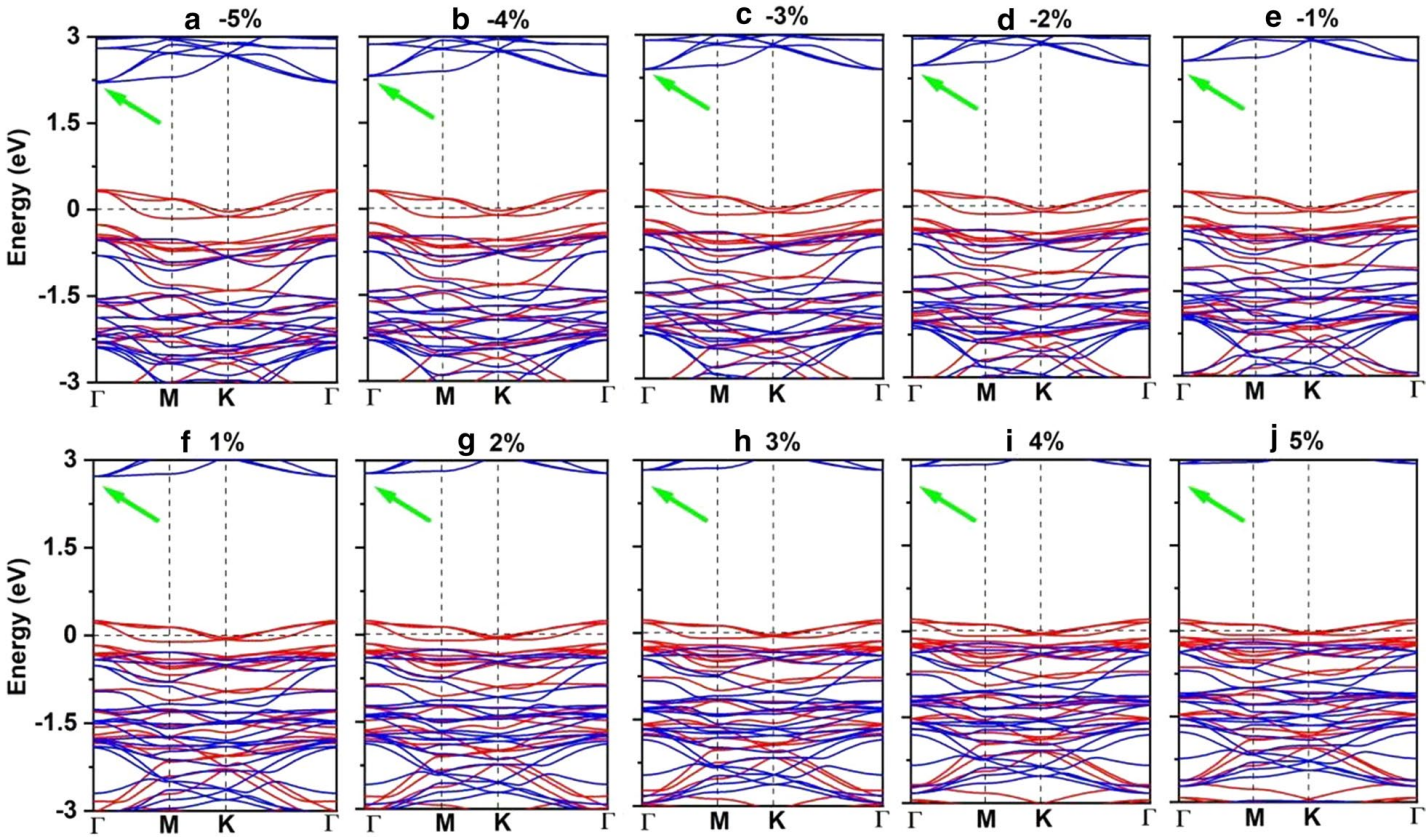
**a**–**j** Spin-resolved band structure for Mn_3_Br_8_ monolayer under biaxial strain from -5% to 5%. The green arrow denotes the indirect band gap

Both the Mn–Mn and Mn-Br distances increase monotonically as the strain changing from –5% to 5%. Correspondingly, the exchange parameter under the biaxial strain presented in Fig. 8a decreases with the biaxial strain changing from –5% to 5% and reach the largest value (1.18 meV) under –5% biaxial strain. The Curie temperature of Mn_3_Br_8_ monolayer under –5% biaxial strain is 160 K (Fig. 9a). Particularly, the Mn-Br bonds under the increasing tensile strain become longer, and the angles of Mn-Br3,4,5,6-Mn deviate from 90°, which are the main reason why the FM super-exchange interaction becomes weaker. Consequently, the Curie temperature decreases. It is similar with CrPTe_3_ and FePS_3_ monolayers [79]. Additionally, the MDE decreases with the increasing strain (Additional file 1: Fig. S5(b)); the MAE under –1% biaxial strain is the largest (–3.04 meV). The –5–5% strain does not cause large structural deformation for Mn_3_Br_8_ monolayer, and the morphology of its band structures hardly changes. Mn_3_Br_8_ monolayer keeps to be half-metallic. Both VBM and CBM in the semiconducting spin-channel move upward slightly to the higher energy as shown in Figs. 8c and 10; the band gap increases slowly with the increasing biaxial strain to 3.12 eV under 5% biaxial strain.

Electron/hole doping always leads to VBM/CBM moving away from the Fermi level. Our calculations show that Mn_3_Br_8_ monolayer with –1–1e (~ $1.7 \times 10^{14} {\text{cm}}^{{ - 2}}$) carrier doping per formula unit is still FM; the atomic magnetic moment of each Mn ion is still 13/3μ_B._ As shown in Fig. 7b and d, with carrier doping from –1e to 1e per formula unit, the Mn-Br-Mn angles involving Br3,4,5,6 atoms are about 90° ~ 98°; the Mn-Br1,2-Mn angles are about 88° ~ 90°. The Mn–Mn and Mn-Br1,2 distances increase with the increasing electron doping. Mn_3_Br_8_ monolayer with 0.2e and 0.4e carrier doping has larger magnetic exchange parameter (Fig. 8a). The Curie temperature at 0.2e electron doping is largest of 140 K (Fig. 9b). Additionally, with –1e ~ 0.2e doping, the MAE is along in-plane directions; the MDE decreases with the increasing electron doping. Under 0.4e doping, the MCE turns to be positive with the value of 0.41 meV per formula unit; the MAE is only 0.01 meV per formula unit with taking the MDE into account (Additional file 1: Figs. S5(a) and (b)). With 0.6e, 0.8e and 1e doping, the PMA (perpendicular magnetic anisotropy energy) is 1.70, 2.42, and 5.13 meV, respectively, large enough for spintronic applications (Fig. 8b).

**Fig. 11:**
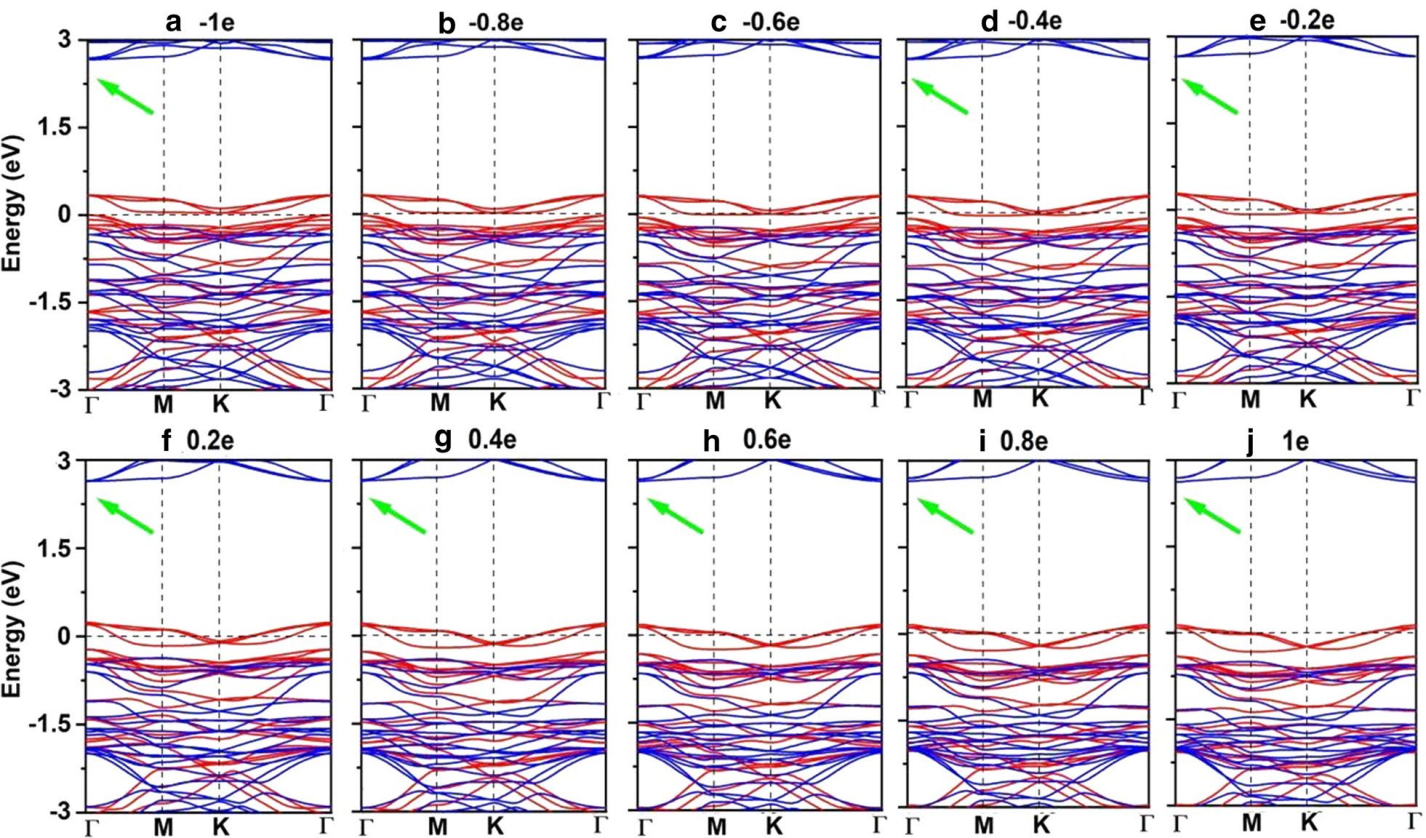
**a**–**j** Spin-resolved band structure for Mn_3_Br_8_ monolayer with carrier doping from -1e to 1e per formula unit. Positive and negative values represent the electron and hole doping, respectively. The green arrow denotes the indirect band gap

Additionally, Mn_3_Br_8_ monolayer with carrier doping of –1e ~ 1e per formula unit maintains to be half-metallic. Its band gap in the semiconducting spin-channel increases/decreases slightly with the increasing electron/hole doping as shown in Fig. 8d; the positions of the VBM and CBM do not change. Exceptional, Mn_3_Br_8_ monolayer turns to be FM spin-gapless semiconductors (SGS) with the metallic spin-channel opening up a very small energy gap (0.07 eV) under –0.6e and –0.8e hole doping; its Fermi level locates in the band gap region (Fig. 11b and c, more clearly figures are presented in Additional file 1: Figs. S6(a) and (b)). Correspondingly, electrons may be easily excited from the valence band to the conduction band with a small input of energy, which simultaneously produces 100% spin polarized electron and hole carriers. The Curie temperature at –0.6e and –0.8e hole doping is 110 K (Fig. 9c and d), higher than liquid-nitrogen temperature (77 K). Considering with that the charge density modulation of $10^{13} \sim10^{15} {\text{cm}}^{ - 2}$ was already achieved experimentally [80–82], our predicted properties of Mn_3_Br_8_ monolayer with carrier doping are also experimentally approachable.

## Conclusions

In summary, the stability, electronic, and magnetic properties of Mn_3_Br_8_ monolayer have been carefully investigated. Our results show that Mn_3_Br_8_ monolayer is FM half-metal with 130 K Curie temperature and with 2.97 eV band gap for the semiconducting spin-channel. Plus, the magnetic moment of each Mn ion is 13/3μ_B_; the MAE is –2.33 meV per formula unit. The Mn_3_Br_8_ monolayer is designed by inducing single Mn vacancy in the ${2} \times {2} \times {1}$ supercell of MnBr_2_ monolayer to break the AFM coupling d^5^ configuration. The feasibility of forming the Mn vacancy and the dynamical, mechanical stability of Mn_3_Br_8_ monolayer have been comprehensively confirmed. Additionally, Mn_3_Br_8_ monolayer under biaxial strain –5% ~ 5% is still FM half-metal with 2.71 ~ 3.12 eV band gap for the semiconducting spin-channel, whose Curie temperature under –5% biaxial strain is 160 K. Both biaxial strain and carrier doping make the MAE increase, which turns to be perpendicular to the plane under electron doping. With 0.8e and 0.6e hole doping, Mn_3_Br_8_ monolayer turns to be spin-gapless semiconductor (SGS) with band gap of 0.07 eV. Our calculations demonstrate Mn_3_Br_8_ monolayer as FM half-metal with high Curie temperature, and having large MAE and large magnetic moment, and tunable electronic and magnetic properties under the applied biaxial strain and carrier doping.

## Supplementary information

**Additional file 1.** Revised supporting information.

## Data Availability

The datasets generated during and/or analyzed during the current study are available from the corresponding author on reasonable request.

## Abbreviations

2D: Two-dimensional
AFM: Antiferromagnetic
CBM: Conduction band minimum
DFT: Density functional theory
DOS: Density of states
FIM: Ferrimagnetic
FM: Ferromagnetic
GGA: Generalized gradient approximation
GKA: Goodenough–Kanamori–Anderson
MAE: Magnetic anisotropy energy
MCE: Magneto-crystalline anisotropy energy
MC: Monte Carlo
MDE: Magnetic dipolar anisotropy energy
MTJ: Magnetic tunneling junctions
NM: Non-magnetic
NN: Nearest neighboring
PAW: Projector augmented wave
PBE: Perdew–Burke–Ernzerhof
PMA: Perpendicular magnetic anisotropy energy
PNGN: Porous nitrogen-doped graphene networks
SGS: Spin-gapless semiconductor
SOC: Spin–orbit coupling
VASP: Vienna ab-initio simulation package
VBM: Valence band maximum
VDW: Van der Waals
