# Supplementary material for: Conversation from antiferromagnetic MnBr2 to ferromagnetic Mn3Br8 monolayer with large MAE
Source: Nanoscale Res Lett. 2021 Apr 29;16:72. doi: 10.1186/s11671-021-03523-0 (PMC8085181; doi:10.1186/s11671-021-03523-0)
Supplement: Supplementary file 1 — Additional file 1. Revised supporting information. [file 11671_2021_3523_MOESM1_ESM.docx]

**Supporting information**

**Conversation from anti-ferromagnetic Mn­Br_2_ to ferromagnetic** **Mn­_3_Br_8_ monolayer with large MAE**

*Y. Hu, ^1^ S. Jin, ^2^ Z. F. Luo ,^1^* *H. H. Zeng,^1^ J. H. Wang^1^, X.L. Fan^1*^*

^1^ State Key Laboratory of Solidification Processing, Center for advanced lubrication and seal Materials, School of Material Science and Engineering, Northwestern Polytechnical University, 127 YouYi Western Road, Xi’an, Shaanxi 710072, China

^2^ Queen Mary University of London Engineering School, Northwestern Polytechnical University, 127 YouYi Western Road, Xi’an, Shaanxi 710072, China

Table S1. The calculated total energies of ferromagnetic (FM), anti-ferromagnetic (AFM), and ferrimagnetic (FIM) configurations relative to the nonmagnetic (NM) configuration for MnBr_2_ and Mn_3_Br_8_ monolayers.

|  | NM | FM | AFM (AFM-1) | FIM | AFM-2 |
| --- | --- | --- | --- | --- | --- |
|  | (eV) | (eV) | (eV) | (eV) | (eV) |
| MnBr_2_ | 0 | -3.193 | -3.913 | - | - |
| Mn_3_Br_8_ | 0 | -9.837 | -9.708 | -9.804 | -9.740 |

Table S2. The calculated elements of 2D elastic constants matrix and the in-plane stiffness for MnBr_2_ monolayer.

|  | $C_{11}$ | $C_{22}$ | $C_{66}$ | $C_{12}$ | $C_{21}$ |  |  |
| --- | --- | --- | --- | --- | --- | --- | --- |
|  | (J∙m^-2^) | (J∙m^-2^) | (J∙m^-2^) | (J∙m^-2^) | (J∙m^-2^) | (J∙m^-2^) | (J∙m^-2^) |
| MnBr_2_ | 29.42 | 29.42 | 10.48 | 8.47 | 8.47 | 26.98 | 26.98 |

**Analysis of the elastic constants and deformation**

The $C_{11}$ and $C_{22}$ are equal to each other, which exhibits the structural and mechanical isotropy; the rigidity against deformation are about three times softer along the c direction comparing with that along the a or b directions. The gravity induced out-of-plane deformation is estimated by the following equation via using the in-plane stiffness [S1]:

$$\frac{h}{L}\approx(\frac{\rho gL}{Y_{2D}})^{1/3}$$

where $\rho=4.91\times10^{-6}\text{Kg/m}^{2}$ is the mass density of MnBr_2_, and $L$ is the size of the monolayer. Taking $L\approx100\text{μm}$, we obtain $\frac{h}{L}\approx5.72\times10^{-4}$, which is comparable to that of monolayer CrI_3_ ($6.45\times10^{-4}$) [47], and is of the same magnitude order of graphene [S1]. Our result shows that the free-standing MnBr_2_ monolayer can withstand its own weight and maintain the planar structure.


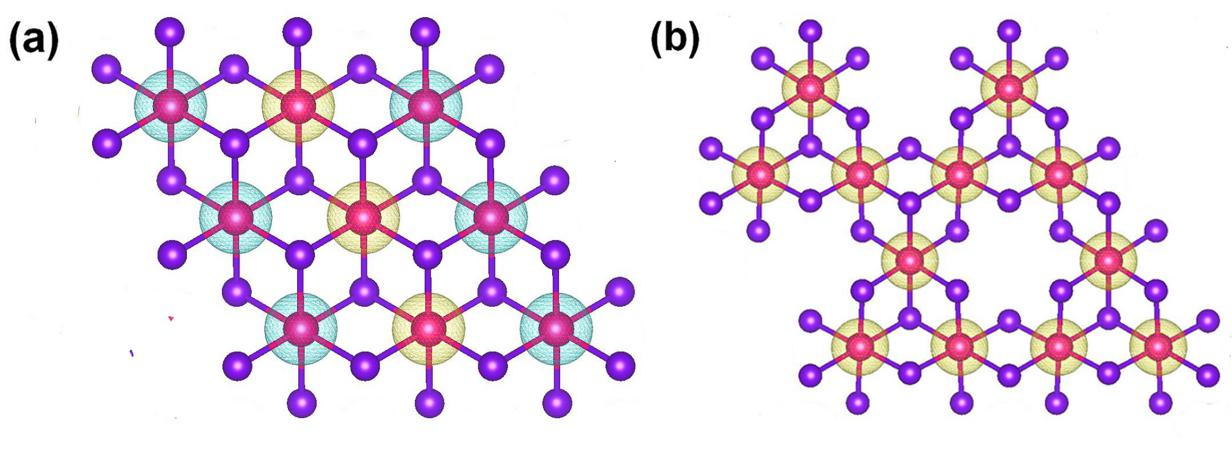


Fig. S1. (a) Spin-resolved charge density (SCD) for (a) MnBr_2_ and (b) Mn_3_Br_8_ monolayers. The isosurface value for SCD is set as 0.014 eÅ^-3^.


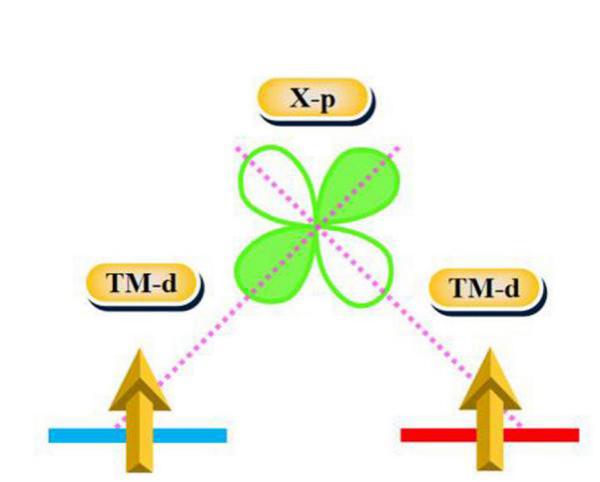


Fig. S2. The ferromagnetic super-exchange interaction according to the Goodenough-Kanamori-Anderson (GKA) rule.


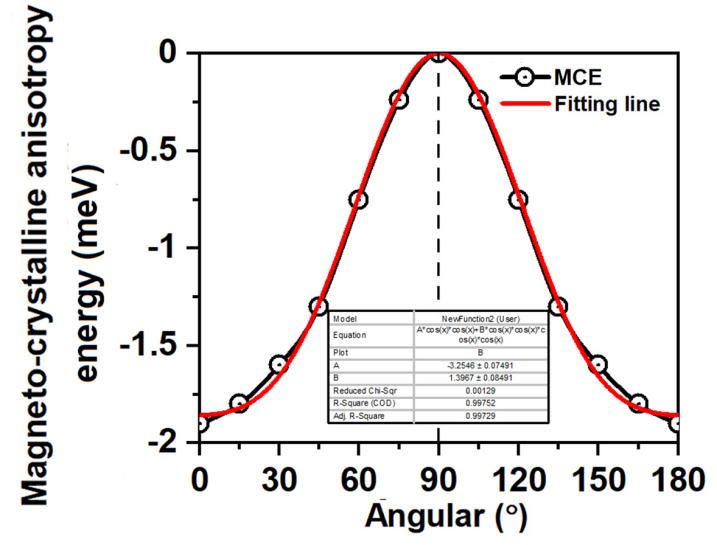


Fig. S3. The variation of magneto-crystalline anisotropy energy (MCE) with respect to azimuthal angle. Red solid line is the fitting line, the inset table lists the standard errors for the fitting slope.


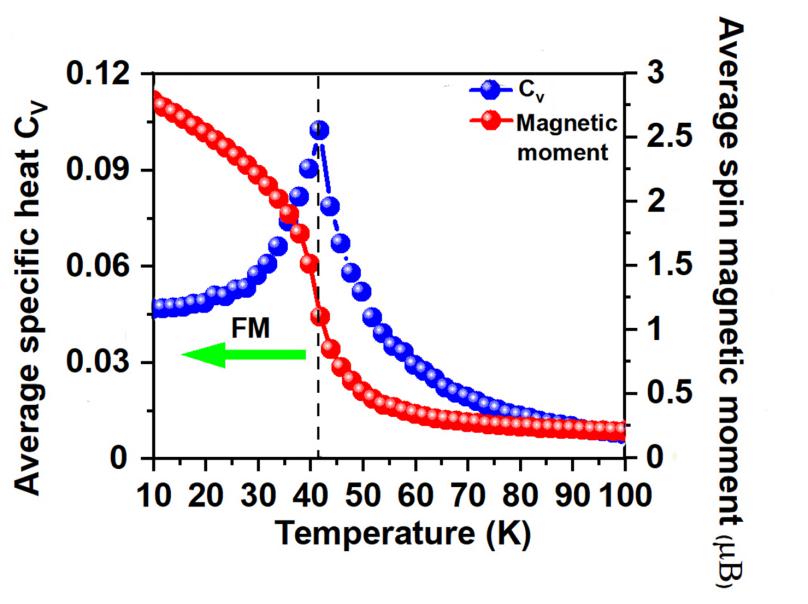


Fig. S4. On-site magnetic moments of Cr atoms and the specific heat $C_{v}$ as function of temperature based on Heisenberg model for CrI_3_ monolayer, the value of $J$ and $A$ are obtained from J. Am. Chem. Soc, 2018, 140, 2417.


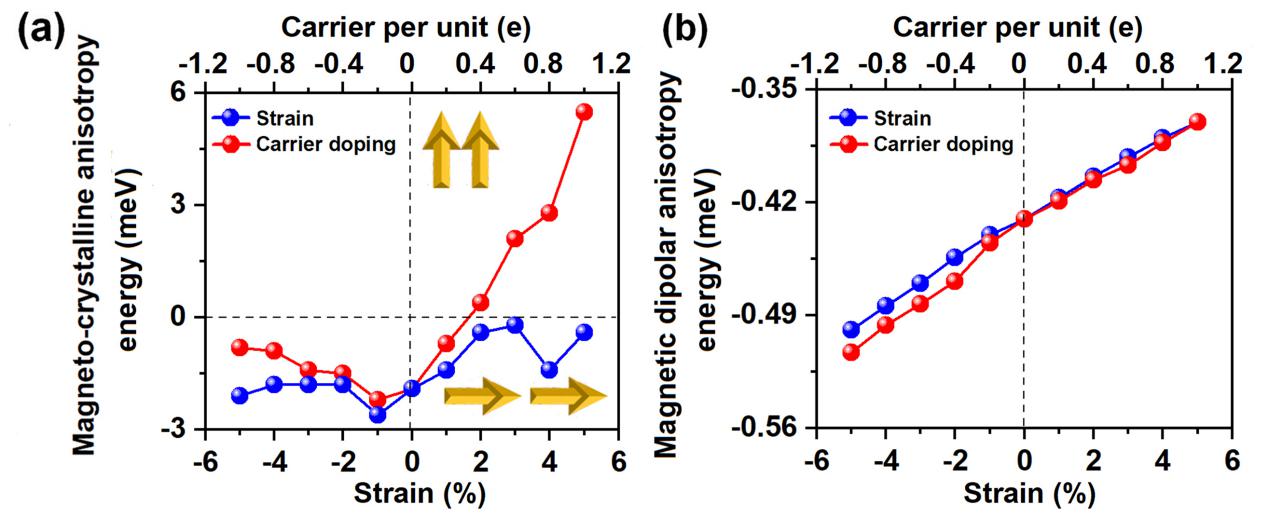


Fig. S5. The variations of (a) magneto-crystalline anisotropy energy (MCE) and (b) magnetic dipolar anisotropy (MDE) with respect to the applied biaxial strain and carrier doping. Positive and negative values of carrier doping represent the electron and hole doping, respectively.


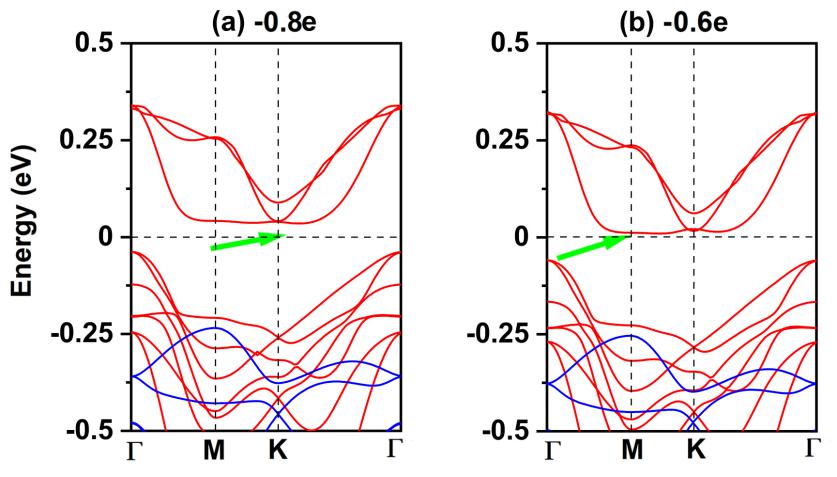


Fig. S6. Spin-polarized electronic band structures for Mn_3_Br_8_ monolayers with carrier doping of (a) -0.8e and (b) -0.6e per formula unit, respectively. The green arrow denotes indirect band gap.

**References**

S1. Booth TJ, Blake P, Nair RR, Jiang D, Hill EW, Bangert U, Bleloch A, Gass M, Novoselov KS, Katsnelson MI, Geim AK (2008) Macroscopic graphene membranes and their extraordinary stiffness. Nano Lett 8:2442.
